# Reference Value of Immunoglobulins in Healthy School Children of Bangladesh

**DOI:** 10.2188/jea.11.263

**Published:** 2007-11-30

**Authors:** Jasimuddin Ahmed, M Mostafa Zaman, Mian Abdur Rouf, M M Monzur Hassan, Salma Zareen

**Keywords:** immunoglobulin, reference value, Bangladeshi children, developing country

## Abstract

The reference value of immunoglobulins (Igs) should be known for a population concerned because it is influenced by many clinical and local conditions. As yet the reference value of the Igs have not been determined in Bangladeshi children. This study determined the reference value of Igs in apparently healthy 261 rural Bangladeshi primary school children (aged 5 to14 years, mean 9.3 years). IgG, IgM and IgA were determined by an auto-analyzer. The mean (standard deviation) value of IgG was 1728 (344) mg/dl. The corresponding values for IgM and IgA were 200 (88) and 163 (63) mg/dl, respectively. The 95% reference value calculation in all subjects showed that the range for IgG was 1103 to 2524, IgM was 92 to 390, and IgA was 72 to 325 mg/dl. These values could be used to evaluate Ig status in children with a variety of clinical conditions.

## INTRODUCTION

Human body is equipped with a special system of defense mechanism. The chief armor of the defense mechanism is immunoglobulins (Ig), which are the effector products of differentiated B-cells and mediate the humoral arm of the immune response. Three main components of the Igs (G, M and A) are measured for many clinical conditions particularly in children. Interpretation of the results depends on the known reference values, and these values vary among populations. Therefore, it is necessary to determine the reference value in the population under question.

In Bangladesh, no report is available on normal level of Igs in children. This study was done to find out the normal level of IgG, IgM and IgA in healthy school children of Bangladesh.

## MATERIALS AND METHODS

Primary education in Bangladesh consists of five years from grades I through V and is free of cost. Usually children enroll in to primary schools at 5 to 6 years of age, but there is no strict rule. This study was done, during February through May 1992, in Sanora Government Primary School located in Sanora village of Dhamrai upazilla (sub-district) in Dhaka district. Children of all socioeconomic status from the nearby area attend this school. It is run by a management committee, which consists of representatives of parents, teachers and government officers. A meeting was held between the investigators and management committee, which also included the local health authority. Consent was obtained from the management committee. Then class teachers explained the purpose of the study to the students and requested them to participate in the study. A total of 450 boys and girls of all grades participated in the study. We excluded those who suffered from fever and sore throat during the last four weeks, and those who had clinical evidence of tonsillo-pharyngitis on physical examination. None of them suffered from immunodeficiency diseases. Thus, 361 (80.2%) children were selected for laboratory investigation. Among them 261 (72.2%) subjects were finally selected for this study after excluding those with C-reactive protein more than 0.05 mg/dl^1^^)^ and yielded bacteria on throat culture (mostly streptococci and staphylococci). Blood samples were collected in fasting condition and sera were separated within two hours of venepuncture and preserved in -80°C until measurement of total protein, albumin, C-reactive protein, IgG, IgM and IgA. Multipurpose Immunochemistry System Autoanalyzer Model LA-2000 (Eiken Chemical Company, Japan) using latex reagent was used to measure Igs. All reagents used in this study were from the same company.

### Statistical analysis:

All statistical analyses were done by using SAS statistical package (Release 6.11, SAS Institute Inc., Cary, NC). The reference value for Igs were defined as the ranges that included 95% of values obtained from the percentile distributions. Results are presented according to age groups because there are some reports from developed countries^2^^)^ that Ig levels in children are related to age.

## RESULTS

Subjects are 5 to 14 years (mean 9.3 years) old, and boys and girls are similar in age. Age-specific levels of proteins are presented in Table 1. Percentile distributions with mean (standard deviation) according to age groups are presented in Table 2. There is no age related trend of immunoglobulin levels. The 95% reference value calculation in all subjects showed that the range for IgG was 1103 to 2524, IgM was 92 to 390, and IgA was 72 to 325 mg/dl.

**Table 1.** Mean (standard deviation) of protein levels by age.

Age, year	Number	Total protein (mg/dl)	Albumin (mg/dl)	Globulin (mg/dl)	Albumin/ Globulin ratio
5 - 7	46	7.5 (0.5)	4.0 (0.2)	3.5 (0.5)	1.2 (0.2)
8	49	7.8 (0.5)	4.2 (0.3)	3.6 (0.5)	1.2 (0.3)
9	47	7.8 (0.7)	4.3 (0.4)	3.5 (0.7)	1.2 (0.3)
10	52	7.8 (0.6)	4.2 (0.3)	3.6 (0.7)	1.2 (0.3)
11	38	7.7 (0.5)	4.2 (0.3)	3.5 (0.4)	1.2 (0.2)
12 - 14	29	7.8 (0.5)	4.3 (0.3)	3.6 (0.5)	1.2 (0.2)
Total	261	7.7 (0.5)	4.2 (0.3)	3.5 (0.6)	1.2 (0.2)

**Table 2.** Mean (standard deviation) and percentile distribution of immunoglobulins (mg/dl).

Age group	Number	Immunoglobulin G				Immunoglobulin M				Immunoglobulin A			
		
		Mean (SD)	2.5th	50th	97.5th	Mean (SD)	2.5th	50th	97.5th	Mean (SD)	2.5th	50th	97.5th
5 - 7	46	1679 (336)	1107	1643	2342	207 ( 98)	72	202	462	155 (59)	81	147	250
8	49	1755 (377)	1224	1660	2636	229 (131)	108	214	721	161 (60)	82	147	283
9	47	1775 (350)	1132	1755	2502	194 ( 72)	103	193	390	165 (59)	72	155	348
10	52	1736 (356)	1090	1714	2551	204 ( 67)	116	188	339	157 (73)	67	144	345
11	38	1683 (352)	930	1652	2426	173 ( 46)	84	173	269	158 (53)	72	158	297
12 - 14	29	1734 (319)	1165	1796	2228	182 ( 65)	92	174	344	188 (71)	57	191	340
Total	261	1728 (344)	1103	1690	2524	200 ( 88)	92	189	390	163 (63)	72	153	325

## DISCUSSION

Estimation of Ig is now an established tool for the study of immune status or the immunologic response of an individual in a high-risk condition. The reference value of Igs should, therefore, be established by using data from a representative local healthy population. To the best of our knowledge, this is the first study of Igs in apparently healthy school children in Bangladesh. The school that we have selected for this study includes children from all socioeconomic background of the locality and may represent primary school children of rural Bangladesh, but not Bangladesh at large. More representative studies are necessary to validate our finding. It could be more informative to know the situation in other developing countries, particularly South Asian countries. Unfortunately, such data for children are not available from those countries.

Maturation of immune responses is a continuous process in childhood. Adult level of IgG reached in the child by the seventh year of life and remains relatively constant thereafter^3^^)^. After birth, the rate of synthesis of IgM increases rapidly and adult level may be reached by the ninth month of age^4^^)^. Some authors^5^^)^ have shown that IgA synthesis begins during the first few weeks after birth and the concentration rises slowly during the first year and continued throughout early adulthood. However, we did not observe any age dependent change in Ig levels, which is similar to the study of Stiehm et al^6^^)^ for IgG and IgM but not for IgA. Higher level of serum Igs in Ethiopian pre-school age children, as compared with the Swedish, has been reported by Johansson *et at*^7^^)^ which is similar to our study. Repeated infection and malnutrition in early life remains the underlying cause of elevated Ig levels in children of developing countries^7^^, ^^8^^)^ including Bangladesh. Children in Bangladesh are often exposed to malnutrition and various type of respiratory and intestinal infections in the early childhood. Therefore, they attain adult level of Igs before adulthood, and age-dependent changes in childhood become masked.

### Possible bias:

To recruit healthy children we have excluded those who had fever during last month, and tested positive for throat swabs and C-reactive protein. This definition for healthy children does not necessarily ensure inclusion of healthy children (and exclusion of sick children).

### Implication:

In absence of a recognized reference range of Ig, determination of immune status would vary among physicians. We believe that the reference values suggested here would reduce such variations and may help in diagnosis and therapeutic decision making. On the other hand, the cut-off points for the reference value on a continuous test result is an arbitrary decision^9^^)^. This is true for any test result that takes on a range of values^10^^)^. Physicians should use their clinical judgement for decision making. As the immunoglobulin distributions are heterogeneous among the population, it is necessary to determine the distribution of Igs for the population concerned. However, in absence of data for the population under question, present data may provide some information for developing countries especially South Asian countries.
